# Hospitals and Poor Law Reform

**Published:** 1910-04-02

**Authors:** H. Russell Wakefield

**Affiliations:** The Deanery, Norwich


*To the Editor of* The Hospital.

### HOSPITALS AND POOR LAW REFORM.

*To the Editor of* The Hospital.

Sir,—Your article on Hospitals and Poor Law Reform is one that deserves the sympathetic consideration of those responsible for the Minority Report of the Royal Commission on the Poor Law. You say rightly that for all practical purposes we may regard the proposals of the Majority in regard to medical relief as having failed to get such support as would give them a likelihood of being brought into effect.

In regard to the Minority scheme, you are anxious to find a *modus vivendi* which will ensure the continuance of the voluntary hospital side by side with a State medical service. Personally I regard this as desirable and not difficult to ensure. Taking our proposals at their severest, only suggest that there is a minimum which the State should accomplish, whilst for the voluntary work in all directions of social welfare there will remain a more satisfactory field of labour than it has at present. I believe the voluntary hospital might have under our Minority scheme untold possibilities for a more unhampered benefiting of individual and general health.

I hope sincerely that we of the Minority Commissioners may have the opportunity of conference with representatives of the voluntary hospitals, for, though I am writing without consulting my colleagues, I am sure we shall do all we can to improve rather than hurt those institutions.

Yours faithfully,

H. Russell Wakefield.

The Deanery, Norwich, March 24.

